# Topical Steroid Withdrawal in Atopic Dermatitis: Patient-reported Characterization from a Swedish Social Media Questionnaire

**DOI:** 10.2340/actadv.v105.40187

**Published:** 2025-01-03

**Authors:** Mikael ALSTERHOLM, Maja AF KLINTEBERG, Sophie VRANG, Gunnthorunn SIGURDARDOTTIR, MariHelen SANDSTRÖM FALK, Alexander SHAYESTEH

**Affiliations:** 1 Division of Dermatology and Venereology, Department of Medicine Solna, Karolinska Institutet, Stockholm; 2 Department of Dermatology and Venereology, Institute of Clinical Sciences, Sahlgrenska Academy, University of Gothenburg, Gothenburg; 3 Department of Public Health and Clinical Medicine, Dermatology and Venereology, Umeå University, Umeå; 4 Patient organisation Atopikerna, the Swedish Asthma and Allergy Association, Stockholm; 5 Department of Dermatology and Venereology in Östergötland, and Department of Biomedical and Clinical Sciences, Linköping University, Linköping; 6 Vasakliniken Dermatology Clinic, Gothenburg, Sweden

**Keywords:** atopic dermatitis, red skin syndrome, topical steroid addiction, topical steroid withdrawal, topical steroid withdrawal syndrome

## Abstract

Topical steroid withdrawal (TSW) is described as an adverse reaction to topical glucocorticoids (TGCs). A pathophysiological mechanism has not been identified. There are no diagnostic criteria. The aim was to describe patient-reported characteristics of TSW in atopic dermatitis (AD). An observational cross-sectional study was performed by posting a questionnaire for participants, aged ≥18 years, reporting both AD and TSW, in a Swedish TSW-themed Facebook group during 4 weeks in 2023. The questionnaire was accessed by 98 participants, with 82 completing it. Most were female (95%), 18–39 years old (74%), self-diagnosed with TSW (84%), and reported current symptoms of AD and TSW. They defined TSW as dependency on TGCs and adverse reactions to their use. Erythema, desquamation, dryness, and oozing affecting the face, neck, and upper extremities were the most reported signs. Pruritus, sleep disturbance, and signs of anxiety and depression were the most reported symptoms. Recurring episodes of manifestations attributed to TSW were reported by 60%. The personal trigger factor was believed to be TGCs by 93%, and 33% also identified oral glucocorticoids. TGCs were currently used by 21%. Self-reported manifestations of TSW are similar to those of AD but appeared to be distinguishable for the participants and caused considerable morbidity.

## SIGNIFICANCE

Topical steroid withdrawal (TSW) is not recognized by healthcare providers because there are limited data to support the proposed mechanism of addiction and withdrawal as an explanation for the patient-reported manifestations. TSW is widely discussed on social media, mainly based on personal accounts from sufferers of TSW and patient advocacy groups. Research is needed to prevent increasing discordance between patient concerns and healthcare provider engagement. This study provides a detailed patient-reported characterization of TSW in atopic dermatitis (AD). Awareness of symptoms attributed to TSW can help healthcare providers improve the management of concerns regarding topical glucocorticoid (TGC) use.

Topical steroid withdrawal (TSW) has attracted increasing attention, and raised considerable concern, among patients in recent years (1). There is no clear definition of TSW, but it is usually described as an erythematous, burning, and/or painful skin reaction following the tapering or discontinuation of topical glucocorticoids (TGCs). Symptoms have also been reported to occur during treatment with TGCs (2). An association with overuse of potent TGCs is suggested (1). The discourse on TSW largely takes place online and on social media and is based on contents provided by patient advocacy groups and those affected (3, 4). Meanwhile, healthcare providers have remained cautious in recognizing TSW as a pathophysiologically distinct reaction to TGCs, as there is a lack of evidence-based information (1, 5, 6). Symptoms attributed to TSW could have multiple explanations, including but not limited to recognized adverse reactions to TGCs, such as acne, perioral dermatitis, or rosacea, treatment of a skin disease unresponsive to TGCs, skin disease of such severity that TGCs are insufficient, or contact dermatitis (7, 8). There are also concerns that online misinformation regarding TGCs can cause steroid phobia among patients who are likely to benefit from treatment without side effects (9, 10).

The prevalence and demographics of TSW are unknown (11). In a review of case series, symptoms attributed to TSW predominantly affected females with prolonged use of potent TGCs on skin areas and against skin conditions where TGCs might not be suitable or safe (for example rosacea, genital dermatoses, skin bleaching) (1). Two clinical subtypes of TSW have been described – papulopustular and erythematoedematous – and they are considered to be adverse effects of TGC misuse (1).

In clinical practice, we sometimes meet patients who express steroid phobia, but rarely encounter individuals who identify themselves as TSW sufferers. Based on the TSW discussion on social media, it can be assumed that affected individuals hesitate to revisit healthcare providers who prescribed TGCs. Patients with symptoms attributed to TSW have reported dismissive behaviour from healthcare professionals when expressing concerns regarding TGCs (12). These circumstances, combined with the lack of diagnostic criteria, make it challenging to define and reach a study population for the investigation of TSW.

Atopic dermatitis (AD) is a prevalent inflammatory skin disease, and patients frequently receive TGCs (13). Experiences of TSW are likely to be found in this population.

We used social media to distribute a digital questionnaire to individuals with AD who had experienced symptoms that they attributed to TSW. The questionnaire aimed to investigate the participants’ own characterization of TSW and their experience of the manifestations.

## METHODS

### Questionnaire design principles

An online questionnaire was constructed in SurveyMonkey^®^ (SurveyMonkey Inc, San Mateo, CA, USA, www.surveymonkey.com) (Appendix S1). SurveyMonkey^®^ is an easy-to-use tool that collects information in a timely fashion (14). The authors constructed the questionnaire in collaboration with the steering group of SwedAD, the Swedish nationwide registry for patients with AD receiving systemic pharmacotherapy (15).

The questionnaire collected demographic data and investigated 2 domains: AD and TSW. Items in the AD domain were intended to confirm an atopic diathesis and to register current AD treatment. Items in the TSW domain covered clinical signs, symptoms, perceptions, healthcare utilization, and impact on health-related quality of life. Items were mostly multiple choice, with the option to provide a free-text answer if the alternatives given were not applicable. There were also open-ended items and items where participants responded on a visual analogue scale (VAS) (16).

For participants’ convenience and to increase the response rate, the response time for the questionnaire was not to exceed 15 min. The questionnaire was automatically terminated if the participant did not meet the inclusion criteria: previous or ongoing AD, and previous or ongoing TSW (Figs S1 and S2).

### Questionnaire construction

A draft with 44 items was constructed and reviewed by the authors. The items were then transferred to SurveyMonkey^®^ for subsequent construction steps. The authors assessed individual items using a dichotomous rating (yes/no), and commented on general aspects and separate items. This yielded a 48-item questionnaire, on which 2 experienced academic dermatologists from the SwedAD steering group gave feedback regarding relevance, comprehensiveness, language, and length. The feedback was considered, and revisions made. The resulting 47-item questionnaire was tested on 10 dermatologists from the SwedAD steering group, leading to minor changes in wording and language.

In the final step, an information letter regarding the study was sent to representatives from the patient organization Atopikerna within the Swedish Asthma and Allergy Association, inviting them to a pilot test of the questionnaire. Six representatives, all patients with AD, completed the questionnaire on electronic devices and evaluated it by answering 4 questions: “1. Were the items relevant for you as an individual with AD?”, “2. Were the items and their corresponding responses presented in a logical sequence?”, “3. How did you perceive the language of the items (spelling, word choices, grammar)? Please suggest improvements”, and “4. Were any of the items ambiguous? If so, please specify and suggest improvements.” Clarification of some items were made based on their feedback, but the number of items was unchanged (*n* = 47).

### Questionnaire distribution

Through Atopikerna, we contacted the administrator of a Swedish TSW-themed private Facebook group with a request to post. The post explained the aim of the study and linked to the questionnaire. Participants were encouraged to share the link via social media to reach other potential respondents with relevant experiences (17). The post was made on 24 April 2023 with reposts (9 May and 17 May) as reminders. The questionnaire was open from 24 April to 21 May 2023.

### Text analysis

A text analysis was performed on the open-ended item “How would you define the term TSW?”. A limited free-text answer was requested. The answers were read several times by authors MaK and AS, who wrote down keywords independently. Topics were constructed with keywords representing common content (18). Keywords and topics were then discussed until consensus was reached. The frequencies of mentioned topics were calculated.

### Statistical analysis

Descriptive statistics were performed using IBM SPSS Statistics for Windows, version 28.0.1.1 (15) (IBM Corp, Armonk, NY, USA). Results were presented as the number observed per category and as a percentage of the total number of observations. The total numbers observed for each item are given in tables and figures, to indicate missing data. Data collected with a VAS were presented as median values with interquartile ranges. An area-proportional Venn diagram was constructed with BioVenn (biovenn.nl) (Nijmegen, The Netherlands) (19).

## RESULTS

A total of 98 individuals accessed the questionnaire, of whom 82 fulfilled the inclusion criteria and completed it (Fig. S2). Most of the participants were female (95%; *n* = 78), and 74% (*n* = 61) were aged 18–39 years. The average level of education was high, with 70% (*n* = 57) reporting post-high school education (compared with 49% for women in Sweden aged 18–39 years, Statistics Sweden 2022). Answers regarding age at onset of AD, atopic comorbidities, and atopic manifestations among family members were consistent with an atopic diathesis (**Table I**). Ongoing AD and TSW at the time of response was reported by 74% (*n* = 61) and 83% (*n* = 68), respectively. Simultaneously ongoing symptoms attributed to AD and TSW were reported by 65% (*n* = 53) (**Table II**).

**Table I:** Background characteristics for 82 participants with atopic dermatitis (AD) and symptoms attributed to topical steroid withdrawal

Characteristic	*n* (%)
Sex	
Male	4 (5.0)
Female	78 (95)
Age distribution:	
18–29 years	29 (35)
30–39 years	32 (39)
40–49 years	8 (9.8)
50–59 years	10 (12)
≥60 years	3 (3.7)
Highest level of education:	
Elementary school	1 (1.2)
High school	23 (28)
Other post-secondary education	5 (6.1)
University <3 years	18 (22)
University ≥3 years	34 (41)
None of the above	1 (1.2)
Age at AD onset:	
<2 years	35 (43)
2–6 years	33 (40)
7–12 years	2 (2.4)
13–17 years	5 (6.1)
≥18 years	7 (8.5)
Current or previous comorbidities:	
Allergic contact dermatitis	38 (46)
Asthma	28 (34)
Allergic rhinoconjunctivitis	57 (70)
Urticaria	33 (40)
Other	17 (21)
No comorbidities	14 (17)
Family history:	
AD	52 (63)
Asthma	39 (48)
Allergic rhinoconjunctivitis	48 (59)
Urticaria	14 (17)
None of the above	11 (13)
Unknown	3 (3.7)

**Table II:** Activity status of atopic dermatitis (AD) and symptoms attributed to topical steroid withdrawal, and treatment for atopic dermatitis, among 82 participants at the time of response

Factor	*n* (%)
Active AD:	
Yes	61 (74)
No	21 (26)
Current AD treatment:	
Topical treatment	
Emollients	38 (46)
Calcineurin inhibitors	8 (9.8)
Glucocorticoids	17 (21)
Oral treatment	7 (8.5)
Subcutaneous injection	11 (13)
Phototherapy	12 (15)
Other	7 (8.5)
Active TSW:	
Yes	68 (83)
No	14 (17)
If no, time of previously active TSW:	
3–6 months ago	2 (2.4)
6–12 months ago	2 (2.4)
>12 months ago	10 (12)
Number of episodes^a^ of active TSW:	
1	33 (40)
2–4	28 (34)
≥5	21 (26)
Active AD and TSW	53 (65)

a An episode of symptoms attributed to topical steroid withdrawal (TSW) was defined as being resolved after 2 months without symptoms.

Of the 82 participants, 84% (*n* = 69) declared that they diagnosed themselves with TSW. In 5% (*n* = 4) of cases, TSW was diagnosed by a healthcare provider. The remaining 9% (*n* = 7) reported a diagnosis from a social media contact, family member, or friend. Those who denied ever contacting a healthcare provider for symptoms attributed to TSW (*n* = 37) reported fear that the medical staff would not recognize the existence of TSW (*n* = 27) and/or have insufficient knowledge (*n* = 26)

**Fig. 1:**
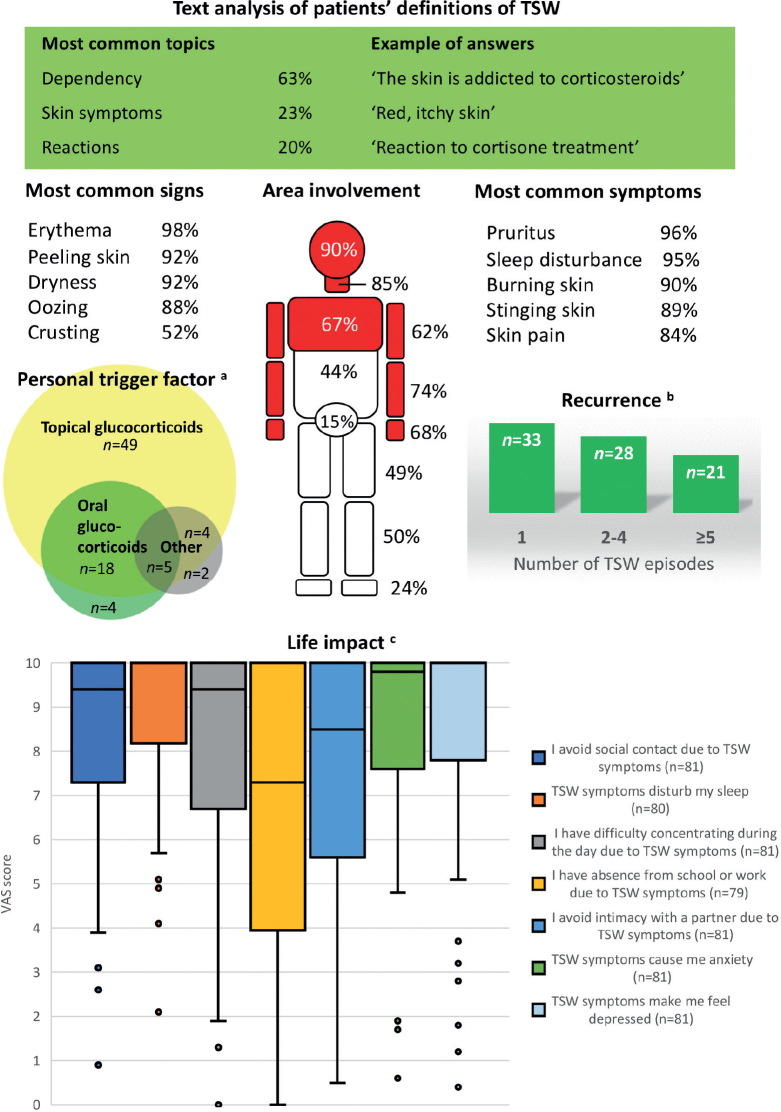
**Main characteristics of self-reported topical steroid withdrawal in participants (*n*=82) with atopic dermatitis.** ^a^Category “Other” distributed over topical calcineurin inhibitor (*n* = 5), other glucocorticoid preparations (*n* = 3), Janus kinase inhibitor (*n* = 1), pregnancy (*n* = 1), and unspecified (*n* = 1). ^b^An episode was defined as being resolved after 2 months without symptoms. ^c^Participants rated applicability to their life from 0 (does not describe my situation at all) to 10 (describes my situation perfectly) on a visual analogue scale. Box-and-whisker plot. The upper quartile equalled the maximum score for all items. TSW: topical steroid withdrawal; VAS: visual analogue scale.

Most of the participants reported recurring episodes of TSW; 2 or more episodes in 60% (*n* = 49) of cases, and 5 or more episodes in 26% (*n* = 21) (Table II, **Fig. 1**). An episode was defined as being resolved after 2 months without symptoms.

The predominant perceived symptoms of TSW were pruritus, sleep disturbance, burning, stinging, and pain from the skin, each reported by more than 80% (Fig. 1). The most common signs were erythema, desquamation, dryness, and oozing from the skin (Fig. 1). Oedema was not included as a separate item response but was infrequently reported in free-text answers. The most commonly affected skin areas were the head and neck and the forearms including the hands (Fig. 1). Frequencies for all reported symptoms, signs, and affected skin areas are available in Fig. S3.

The personal trigger factor for symptoms attributed to TSW was believed to be TGCs by 93% (*n* = 76), but 33% (*n* = 27) also identified oral glucocorticoids (OGCs) as a trigger (Fig. 1). Only 5% (*n* = 4) stated that OGCs, but not TGCs, were their trigger. Additional triggers were reported by 13% (*n* = 11) (Fig. 1).

Many participants had ongoing topical treatment for AD, and 21% (*n* = 17) were using TGCs (Table II). Among TGC users, 15 participants reported ongoing symptoms attributed to TSW. The proportion of participants who defined TSW as dependency on/addiction to TGCs was similar for current TGCs users and those who did not use TGCs, 65% and 60%, respectively. Systemic pharmacotherapy was reported by 22% (*n* = 18), distributed over dupilumab (*n* = 11), methotrexate (*n* = 4), and upadacitinib (*n* = 3). Phototherapy was ongoing for 15% (*n* = 12).

The item “How would you define the term TSW?” was answered by 79 participants with definitions ranging from 5 to 293 characters including spaces. Text analysis resulted in 6 topics where “Dependency”, representing addiction to glucocorticoids, was the most frequent. “Skin symptoms” and “Reactions” were the second and third most frequent topics, illustrating definitions based on symptoms caused by glucocorticoids (**Table III**, Fig. 1).

**Table III:** Text analysis of participants’ definition of topical steroid withdrawal

Definition of TSW	*n* (%)	Example of answers
Topic 1: Dependency	50 (63)	“The skin is addicted to corticosteroids”
Topic 2: Skin symptoms	18 (23)	“Red, itchy skin”
Topic 3: Reactions	16 (20)	“Reaction to cortisone treatment”
Topic 4: Emotional response	8 (10)	“Anxiety, no sleep”
Topic 5: General symptoms	7 (8.9)	“Difficulty regulating body temperature, a burning feeling”
Topic 6: Long-term symptoms	5 (6.3)	“Comes and goes during an undefined time span”

Frequency of mentioned topics with examples of answers to the item “How would you define the term TSW?” Participants (*n* = 79) could mention more than 1 topic.

TSW: topical steroid withdrawal.

The participants were presented with 7 items describing how TSW might affect social interaction, activities, and mood, and asked to rate the applicability of each statement to their own circumstances on a VAS. Considerable impact on sleep, anxiety, and depression was reported (Fig. 1).

## DISCUSSION

In this study, we aimed to define TSW through the experiences of questionnaire responders. The objective was to gather information from as many individuals as possible in a hard-to-define population with a social media presence. Facebook is established as a recruitment tool for connecting with hard-to-reach populations (20). We launched an online questionnaire through a post in a Swedish TSW-themed Facebook group. The link to the questionnaire could be reposted or forwarded, for recruitment of additional responders (17, 21).

We describe a young, predominantly female, self-diagnosed cohort with significant negative life impact attributed to TSW. Most participants reported sleep loss and signs of anxiety and depression. Including emotional impact as part of a definition of TSW could be considered. The skin signs were most common on the head and neck, forearms, hands, and chest. The previously described hand-sparing “red sleeve” distribution was not evident, as the forearms and hands were similarly involved (22, 23). Involvement of the hands is common in adults with AD, which might obscure the presumed TSW phenotype. Systematic reviews have reported mainly facial, and some genital, involvement. Our participants described a wider distribution, possibly reflecting that we investigated an AD cohort (1, 5). Erythema was reported by 98%, but oedema was rarely described, so the erythematoedematous presentation was not clearly represented (1).

The skin manifestations of AD are well documented (24). While the description of clinical signs and symptoms of AD and TSW seem to overlap, 65% of the participants reported activity of both conditions in parallel, indicating that they could discriminate between them. Further study of this distinction should be important when defining criteria for TSW as a potentially particular reaction to TGCs.

Unexpectedly, 21% of the participants reported ongoing TGC treatment despite TSW manifestations, although resumption of use has been reported in a TSW cohort (23). The participants who defined TSW as dependency on TGCs were not more likely to report ongoing TGC use. There were unfortunately no items which investigated this further – for instance, if the participants tolerated some preparations, used TGCs in a deliberate tapering scheme, or lacked better options. A study of TGC treatment habits among affected individuals could increase the understanding of symptoms attributed to TSW. In this context, it is noted that while TSW has previously been described as a one-time occurrence following discontinuation of TGCs, a majority of participants in this study reported several episodes of TSW (1, 5, 11, 22). An episode was defined as being resolved after 2 months without symptoms, so the answers suggested that the participants experienced symptoms, with intermittent remissions, over long periods of time. Unfortunately, no items on whether recurrence was associated with re-exposure to TGCs were included.

A large proportion of the participants reported allergic contact dermatitis (46%; *n* = 38). A limitation of the study is that there were no items investigating which substances the participants were allergic to. Contact allergy against TGC preparations has been suggested as a cause of symptoms attributed to TSW and could be of particular significance in the context of recurrence (7, 8). Only 16% (*n* = 13) reported a contact allergy test prompted by symptoms attributed to TSW.

Interestingly, a third of the participants reported OGCs as a personal trigger factor, in most cases in addition to TGCs. Symptoms attributed to TSW were reported to appear more rapidly with OGCs than with TGCs. The reason for this is unclear. OGCs are not a preferred or recommended treatment for AD and are typically administered for much shorter periods of time than TGCs. It is possible that the high proportion of participants with experience of OGCs in our AD cohort reflected poor symptom control. The proportion of participants reporting OGCs as a trigger factor might have been different if the questionnaire had not selected for AD.

In recent years, several new systemic pharmacotherapies for moderate-to-severe AD have emerged. Despite describing severe symptoms attributed to TGCs, together with ongoing AD, only a small fraction of the participants in this study reported systemic treatment. Dupilumab was the most frequently prescribed systemic drug. Phototherapy was also reported. Systemic pharmaco-therapy could be considered if severe symptoms attributed to TSW arise in patients with AD and has been described to be successful with dupilumab (25). However, most of the participants on systemic pharmacotherapy for AD still reported ongoing symptoms attributed to TSW.

A recent survey on cumulative effects of glucocorticoid exposure in patients with eczema in the United States demonstrated that 75% of the 1,702 respondents reported symptoms consistent with TSW after 3–5 years of TGC use (26). Other authors have pointed out that this result needs to be interpreted with caution as the population was self-diagnosed, part of a TSW support group, and no diagnostic criteria for TSW have been established (27). The respondents to the survey were presented with a definition of TSW that, in addition to describing symptoms attributed to TSW, could apply to the experiences of an individual with AD who discontinues use of TGCs after inadequate treatment response.

In contrast, we asked our participants how they defined TSW. TSW was regarded as an addiction to glucocorticoids, and not limited to topical preparations. TSW was also defined by the bodily reactions to the use and discontinuation of glucocorticoids. The reactions were skin-derived, but systemic and emotional responses were commonly described as well. The reactions following discontinuation were interpreted as a sign of withdrawal of glucocorticoids, distinct from a flare of the underlying AD. These symptoms were seen as dependency – not in the sense of needing treatment, but in the sense of induced addiction to a prescribed substance that resulted in a new set of symptoms, perhaps worse than the initial ailment.

An inherent limitation of the study design was that a response rate could not be calculated; the number of eligible Facebook group members was unknown to us, as was the dissemination of the questionnaire. Potential selection biases include age- and sex-differentiated social media habits, level of education, and the possible correlation between severity of symptoms, social media activity, and motivation to fill out the questionnaire. As the number of participants was relatively small, the results must be interpreted with caution, and the external validity of the study is unknown. However, we believe that we would have reached fewer participants by recruitment through healthcare providers or other modes of advertising.

In the questionnaire design we opted to ask the participants for a free-text definition of TSW, while the items regarding symptoms and life impact were multiple choice or based on the VAS. This strategy aimed to provide a user-friendly questionnaire with a high completion rate, but it can also cause important topics to be missed.

Also, all data were self-reported, meaning that the AD diagnosis was not confirmed by physical examination. We included several items covering AD and atopic comorbidities and found the participants’ answers to be consistent with an atopic diathesis. The lack of a severity rating for the participants’ AD is another limitation.

TSW as a distinct pathophysiological reaction to TGC discontinuation is yet to be confirmed or excluded. This study presents data that can hopefully contribute to the characterization of TSW, as it is experienced and interpreted by those affected. It introduces new aspects such as the recurring episodes of self-reported symptoms attributed to TSW, the continued use of TGCs despite symptoms, and the differentiation of simultaneous symptoms of TSW and AD. These aspects require further research, for instance with qualitative methods. Also, compared with systematic reviews based on case reports and series, this study shows a partly different reported phenotype with regard to the skin signs and their distribution, and includes emotional factors.

As of now, TSW remains an exclusion diagnosis after careful ruling out of established causes for adverse reactions to TGCs. This stance requires that the patients’ concerns and beliefs are respected by empathic and open-minded healthcare providers who uphold an evidence-based approach and offer alternatives to TGCs when possible. When symptoms attributed to TSW arise, a thorough investigation is presumably of great benefit for patients and healthcare providers but requires mutual trust and perseverance. Healthcare providers must continue to rely on the evidence and accumulated experience of TGCs as a safe and effective treatment for most AD patients and at the same time acknowledge that they may not be suitable for everyone.

## Supplementary Material

Topical Steroid Withdrawal in Atopic Dermatitis: Patient-reported Characterization from a Swedish Social Media Questionnaire

Topical Steroid Withdrawal in Atopic Dermatitis: Patient-reported Characterization from a Swedish Social Media Questionnaire
