# Supplementary material for: Topical Steroid Withdrawal in Atopic Dermatitis: Patient-reported Characterization from a Swedish Social Media Questionnaire
Source: Acta Derm Venereol. 2025 Jan 3;105:40187. doi: 10.2340/actadv.v105.40187 (PMC11697140; doi:10.2340/actadv.v105.40187)

Supplementary material has been published as submitted. It has not been copyedited, or typeset by Acta Dermato-Venereologica

Fig. S1. Flowchart for questionnaire items. STOP represents automatic termination of the questionnaire when the chosen item response does not meet the inclusion criteria: previous or ongoing atopic dermatitis and previous or ongoing topical steroid withdrawal. A yellow box represents an item where the chosen response determines the subsequent item.

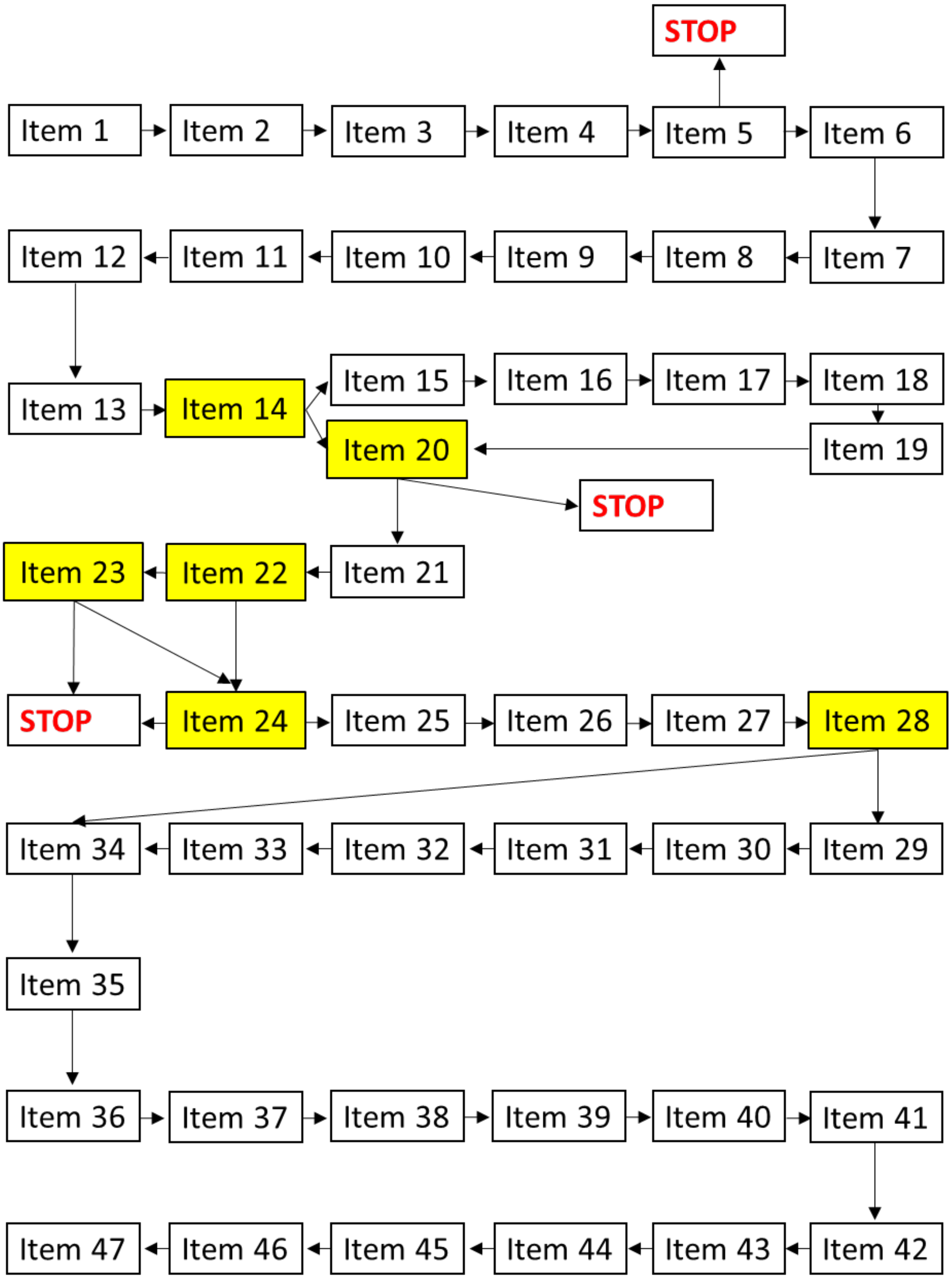

Fig. S2. Flowchart for questionnaire participation. The questionnaire was automatically terminated if a participant did not indicate previous/ongoing atopic dermatitis or previous/ongoing topical steroid withdrawal. There were no answers to topical steroid withdrawal-related items in the incomplete questionnaires (*n*=17). AD: atopic dermatitis; TSW: topical steroid withdrawal.

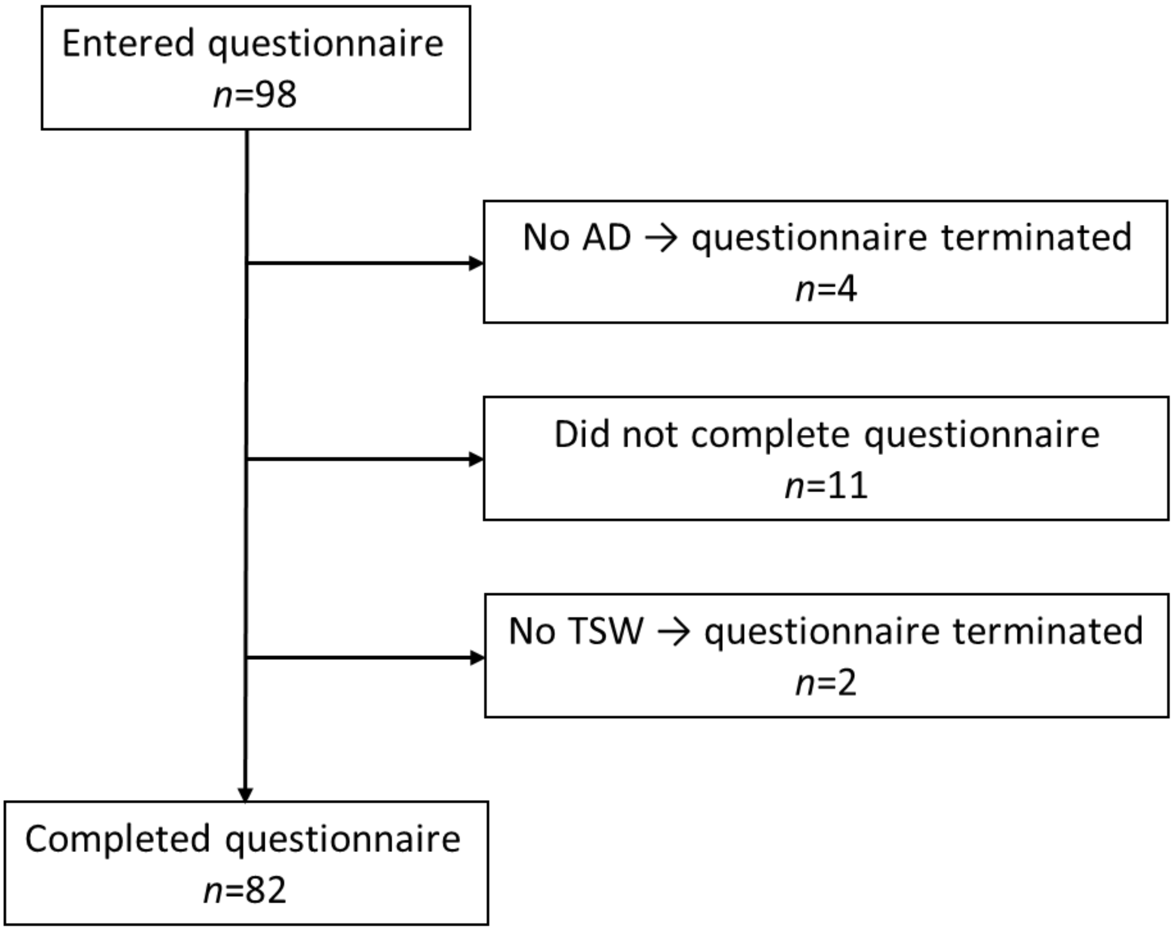

Fig. S3. Frequency of symptoms, clinical signs, and distribution of signs and symptoms in participants with atopic dermatitis and symptoms attributed to topical steroid withdrawal. The participants (*n*=82) were asked which symptoms (panel a) and signs on their skin (panel b) they associated with topical steroid withdrawal based on their current or most recent episode. The bodily distribution of the signs and symptoms is shown in panel c.

a.

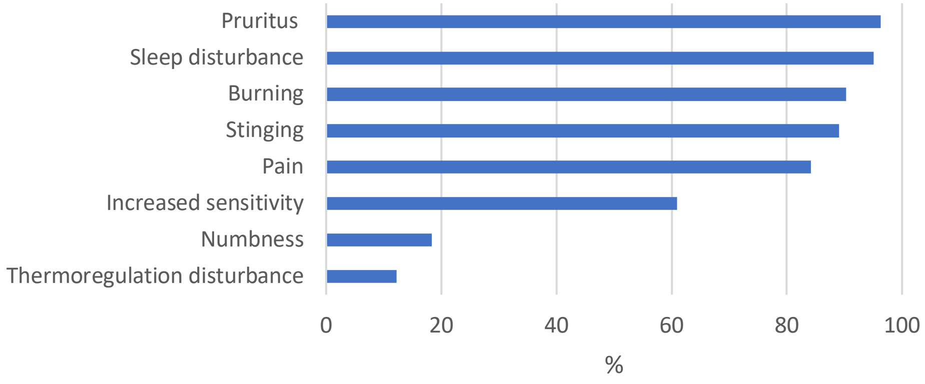

b.

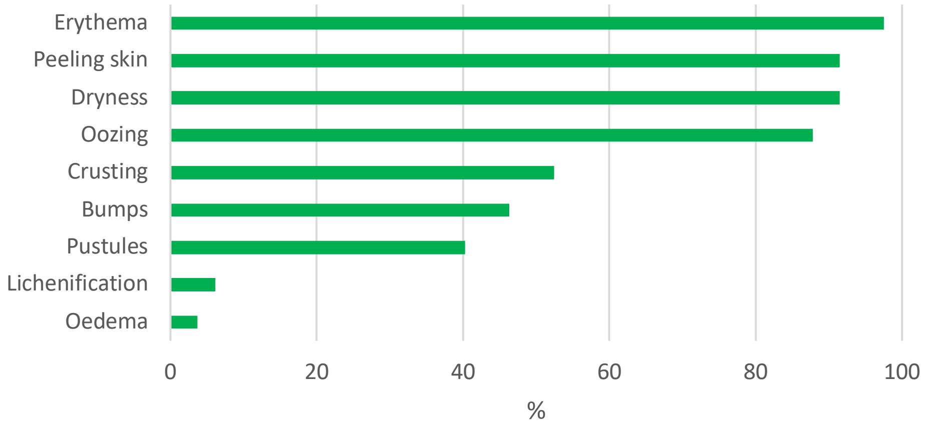

c.

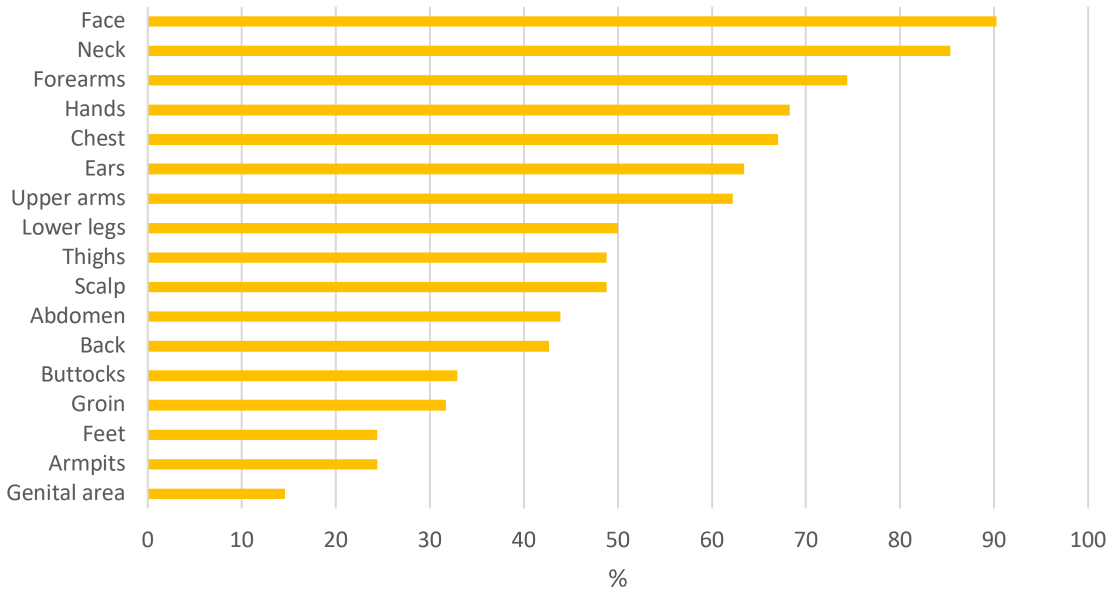

Supplement: Topical Steroid Withdrawal in Atopic Dermatitis: Patient-reported Characterization from a Swedish Social Media Questionnaire [file ActaDV-105-40187-s2.pdf]
